# The acute effect of a β-glucan-enriched oat bread on gastric emptying, GLP-1 response, and postprandial glycaemia and insulinemia: a randomised crossover trial in healthy adults

**DOI:** 10.1186/s12986-024-00789-w

**Published:** 2024-03-18

**Authors:** Ingrid Revheim, Simon Ballance, Adelheid Fretland Standal, Anne Rieder, Jutta Dierkes, Anette E. Buyken, Odd Helge Gilja, Trygve Hausken, Hanne Rosendahl-Riise

**Affiliations:** 1 Centre for Nutrition, Department of Clinical Medicine, Faculty of Medicine, University of Bergen, Bergen, Norway; 2 Norwegian Institute for Food, Fisheries and Aquaculture Research, Ås, Norway; 3 Mohn Nutrition Research Laboratory, Department of Clinical Medicine, University of Bergen, Bergen, Norway; 4 Department of Medical Biochemistry and Pharmacology, Haukeland University Hospital, Bergen, Norway; 5 Institute of Nutrition, Consumption and Health, Faculty of Natural Sciences, Paderborn University, Paderborn, Germany; 6 National Centre for Ultrasound in Gastroenterology, Haukeland University Hospital, Bergen, Norway; 7 Department of Clinical Medicine, University of Bergen, Bergen, Norway

**Keywords:** Oats, β-glucans, Gastric emptying, Ultrasound, Postprandial responses, Glycaemia, Insulinemia, Gastrointestinal hormones

## Abstract

**Background:**

The cereal fibre β-glucan reduces postprandial glycaemia, however, the underlying mechanisms are not fully understood. Thus, the aim of this study was to investigate the acute effect of a β-glucan-enriched oat bread on gastric emptying half-time (*T*_*1/2*_), gastric emptying lag phase (*T*_*lag*_), and gastric emptying rate (GER), and the secretion of glucagon-like peptide-1 (GLP-1) as potential means to influence postprandial glycaemia.

**Methods:**

A randomised crossover trial was conducted in 22 healthy adults (age 24.6 ± 3.1 years, BMI 23.1 ± 2.7 kg/m^2^) receiving 25 g available carbohydrates from a β-glucan-enriched oat bread or a control whole-wheat bread at two non-consecutive days. *T*_*1/2*_, *T*_*lag*_, and GER were determined based on ultrasound measures of the cross-sectional gastric antrum area in the fasting state and 15, 30, 45, 60, 90, and 120 min postprandially. Capillary glucose, serum insulin, and plasma GLP-1 concentrations were measured at the same time points.

**Results:**

A biphasic pattern of gastric emptying with a distinct *T*_*lag*_ before the commencement of emptying was observed in most subjects for both bread types. While no differences in GER were evident (*p* = 0.562), consumption of the oat bread significantly increased *T*_*1/2*_ by 18 min and *T*_*lag*_ by 14 min compared with the whole-wheat bread (*p* = 0.005 and *p* = 0.010, respectively). In addition, the oat bread significantly reduced iAUC_2h_ for glucose and insulin responses compared with the whole-wheat bread (*p* = 0.001 and *p* < 0.001, respectively). There were no significant differences in GLP-1 response between the two breads (*p* = 0.892).

**Conclusion:**

The increased *T*_*1/2*_ and *T*_*lag*_ could offer a potential mechanism for the observed attenuation of postprandial glycaemia and insulinemia after consumption of the β-glucan-enriched oat bread compared with the whole-wheat bread.

*Trial registration*: The study is registered at clinicaltrails.gov (NCT04571866).

**Supplementary Information:**

The online version contains supplementary material available at 10.1186/s12986-024-00789-w.

## Introduction

It is well established that the cereal fibre β-glucan reduces postprandial glycaemia without disproportionally increasing the insulinemic response [1, 2]. Though the viscous properties of β-glucans are thought to be essential for glycaemic attenuation, the precise mechanism is not fully understood. Several mechanisms have been proposed including delayed or reduced starch degradation [3], delayed glucose absorption [4, 5], and changes in gastrointestinal hormone regulation [6]. Further, delayed gastric emptying, which is regarded as a major determinant for an attenuated glycaemic response [7], may also be one of the mechanism responsible for the benefits of viscous dietary fibres, including β-glucans [6].

Gastric emptying can readily be assessed through ultrasonography [8], which is a non-invasive, widely available, and validated method to assess gastric motility, meal accommodation, and gastric emptying [9–11]. Parameters including gastric emptying half-time (*T*_*1/2*_), gastric emptying lag phase (*T*_*lag*_), and gastric emptying rate (GER) are commonly used when assessing gastric emptying. *T*_*1/2*_ is the time to empty half of the stomach content, whereas *T*_*lag*_ reflects the time needed by the stomach to degrade the ingested solids into particles small enough to pass the pylorus [12]. Thus, emptying the stomach of solid food shows a biphasic pattern where a lag phase, during which little emptying occurs, is followed by an exponential phase where solids are emptied [13]. Gastric and duodenal factors such as presence of chyme in the duodenum, energy intake, and digesta viscosity are thought to regulate the process of gastric emptying along with the release of gastrointestinal hormones [12].

The viscous properties of β-glucans have been suggested to increase the transit time in the small intestine and decrease the rate of nutrient absorption [14], increasing the contact time with enteroendocrine cells, and thus increase secretion of gastrointestinal peptides such as glucagon-like peptide 1 (GLP-1) [15]. By supressing the release of glucagon and stimulating β-cells to secrete insulin, GLP-1 influences the postprandial glycaemic responses [15]. Along with other gastrointestinal hormones, GLP-1 is also thought to influence gastric emptying through an intricate neural network [15, 16]. However, the evidence is unclear as the effect of viscous dietary fibres on gastrointestinal hormones is inconsistent across studies [14, 17–19].

To date, studies investigating the effect of β-glucans on gastric emptying show inconclusive results. Increased *T*_*1/2*_ and *T*_*lag*_ after consumption of β-glucan-containing meals compared with meals without β-glucans, but also compared with depolymerised β-glucans (e.g., β-glucans with lower molecular weight (MW)), have been shown [20, 21]. Contrary, though oat flakes and β-glucan-enriched muesli were found to reduce postprandial glycaemia, no difference in gastric emptying were detected following their consumption [22, 23]. Additionally, food structure and food matrix, including interaction between nutrients and non-nutrients that influence the behaviour of food components, might be of importance for β-glucan’s effect on postprandial glycaemia. Oat flakes of different particle size (milled versus whole flake), containing the same amount of β-glucan with similar MW, have been demonstrated to influence postprandial glycaemia, however, not as a function of GER [24].

Taken together, β-glucan’s physiological effect on postprandial glycaemia is evident [25–27]. However, demonstrating the mechanisms responsible for the beneficial effect has been, and continues to be, challenging given the complex physicochemical properties of β-glucans and the differences in food matrixes in which they are applied. The aim of the present study was to explore parameters of gastric emptying and GLP-1 response as factors that potentially contribute to the glucose-lowering effect of β-glucans added to bread. Bread was chosen as stimuli as this is a suitable and realistic vehicle for dietary fibre, it is highly consumed, and it contributes to a significant share of the dietary fibre intake [28]. Gastric emptying *T*_*1/2*_ is the primary parameter. Gastric emptying *T*_*lag*_, GER, and postprandial GLP-1 response are assessed as secondary parameters. An additional secondary objective was to confirm the expected reduction in postprandial glycaemia and insulinemia after consumption of the β-glucan-enriched oat bread compared with the whole-wheat bread.

## Methods

A single-blinded, randomised controlled crossover trial was initiated at the Centre of Nutrition and conducted at the Research Unit for Health Surveys, University of Bergen, Norway. Participants received a β-glucan-enriched oat bread and a control whole-wheat bread on two non-consecutive days with a wash-out period of at least three-days. Healthy males and females aged 18–40 years with a BMI of 18.5–30 kg/m^2^ showing fasting blood glucose concentrations ≤ 7 mmol/L and HbA1c ≤ 48 mmol/mol were eligible. Exclusion criteria were the presence of any acute or chronic disease, current smoking, pregnancy, lactation, food allergies, hypersensitivity or intolerances to wheat or oat, and use of medication affecting glucose tolerance or gastric emptying such as corticosteroids.

All study participants provided written informed consent prior enrolment. The study protocol was approved by the Regional Committees for Medical and Health Research Ethics (ref. 120,533), registered at clinicaltrails.gov (NCT04571866), conducted in accordance with the Declaration of Helsinki, and in consistence with the guidelines for Good Clinical Practice.

### Recruitment and screening

The participants were recruited through advertisements in social media and oral presentations provided to students at the Faculty of Medicine, University of Bergen, Norway. The recruitment took place from August 27 until November 21, 2020. A pre-screening to determine eligibility was conducted through phone interviews. Eligible subjects were invited to attend the study visits at the research unit.

### Randomisation and blinding

The order of the two interventions was randomly allocated using a randomisation scheme stratified by sex and performed in block sizes of four. Subjects were assigned to sequences in the order they attended the first visit. The participants were blinded to the allocation process as well as to the composition of the two breads. Those preparing and serving the test meal, and collecting clinical data, were not blinded as the bread could be identified. The test meal identity was indicated by letters on the sampling tubes and in the case-report forms, so those analysing the samples were unaware of the treatment.

### Study intervention

The intervention bread was an oat bread enriched with oat-derived β-glucan (referred to as oat bread), whereas a whole-wheat bread matched in fat and starch was used as control (referred to as wheat bread). The ingredients for the two study breads are shown in Additional file 4: Table S1 and the methods for estimating nutrient compositions are described in Additional file 1: Methods 1. The two study breads were developed and produced by the Norwegian Institute for Food, Fisheries and Aquaculture Research. The baking process ensured the oat bread to contain β-glucan with a high weight-average MW [29].

The nutrient compositions of the study breads are shown in Table 1. The portions of bread were standardised to provide 25 g available carbohydrates, corresponding to 94.1 g and 60.1 g of the oat bread and the wheat bread, respectively. The standardised serving of oat bread had a higher weight due to a larger content of water than the wheat bread. Though matched in starch and fat, the oat bread contained approximately 12% (83 kJ) more energy and 3.5 g more protein than the wheat bread. The breads were not matched in protein because of a lack of commercially available protein ingredients. Further, the difference in amount was small and, thus, the physiological effect to be expected was deemed minimal.

**Table 1:** Energy content and nutritional composition of the standardised servings of a β-glucan-enriched oat bread and a whole-wheat bread

Per standardised serving	Oat bread (94.1 g)	Wheat bread (60.1 g)
Energy (kJ (kcal))	683.0 (159.5)	599.4 (141.0)
Energy density (kJ/g (kcal/g))	7.3 (1.7)	10.0 (2.3)
Fat (g)	2.9	3.1
Protein (g)	7.6	4.1
Total starch (g)	22.0	21.4
Digestible starch (g)	21.6	21.1
Resistant starch (g)	0.4	0.3
Sugar (g)	1.1	1.6
Total fibre (g)	5.5	2.4
Total β-glucan (g)	3.6	0.2
Moisture (g)	50.7	25.6
Ash (g)	1.8	1.0
Weight-average molar mass of β-glucan (kDa)	1050	N.A

*NA* not analysed

Participants attended two study visits at 8:15 a.m. after an overnight fast. They were instructed to avoid rigorous physical activity 24 h prior the visit, avoid use of nicotine-containing substances during the fasting period, and to maintain dietary habits throughout the study period. Participants were instructed to spend no more than 10 min consuming the bread and the 250 mL of water that was served with the standardised meal, and they were instructed to chew properly.

Blood pressure, body weight, height, and waist circumference were measured according to standardised procedures further described in Additional file 2: Method 2. Blood samples were collected, and gastric antrum cross-sectional area (CSA) measured in the fasting state (0) and 15, 30, 45, 60, 90 and 120 min after initiation of consuming the standardised meals. Baseline measurements were defined as the clinical data collected in the fasting state (t = 0 min).

### Assessment of gastric emptying

The gastric emptying profile of the two breads was determined by real-time two-dimensional ultrasonography using a Sonosite Titan (Sonosite, Bothell, WA) with a 5–2 MHz transducer. The antrum CSA, in cm^2^, was determined using a standardised section where the gastric antrum, the mesenteric vein, and the aorta were visualised. The outer profile of the gastric antrum area was measured using a built-in calliper and calculating program in the instrument. Participants were instructed to be in a seated position slightly leaning backwards, and the readings were conducted when the participants were suspending their breath in expiration, between antral contractions, and by applying minimal force to avoid compression of the antrum. Two scans were taken at each time point, and the antrum CSA was measured twice in each scan. The mean of the four readings was used as an estimate of the antrum CSA. The variation in the measurements was modest and the coefficient of variation values were comparable between the two researchers obtaining the measures, indicating consistent variability in their data collection (coefficient of variation of 6% vs. 8%).

The antrum CSA was used to calculate the gastric emptying parameters *T*_*1/2*_, *T*_*lag*_, and GER. The maximum increase in antrum CSA was calculated by subtracting that measured at fasting from that shortly after the end of the meal (first measuring point at 15 min), according to the method described in Darwiche et al. [30]. This value was designated to have a volume fraction of 1 (i.e. maximum meal volume remaining in the stomach). The antrum CSA of the meal for subsequent time points was determined in the same way by subtracting the fasting value and then calculating the fraction of the meal remaining relative to the maximum. Time zero was defined as the point at which meal ingestion begins [31]. The emptying curves for each subject were fitted to a modified Elashoff power exponential equation y(t) = 1-(1-exp^−kt^)^β^ appropriate for solid-like foods [13] by non-linear regression in SigmaPlot 12.5 (Systat Software, San Jose, CA). y(t) is the fraction of meal remaining in the stomach at time t, β is the extrapolated y-intercept from the terminal portion of the curve and k is the GER in min^−1^. The gastric *T*_*½*_ can be calculated using y(t) = 0.5 and solving for t, *T*_1/2_ = (− 1/k) · Ln(1 − 0.5^1^/β). For solids, the initial delay portion of the curve is often characterized by a lag-phase index, *T*_*lag*_. This is numerically equal to In β/k and is the time in minutes when the second derivative of the function is equal to zero.

### Biochemical analyses

Capillary blood samples were collected by finger-pricks. A catheter was inserted into the antecubital vein for collection of venous blood. The methods used to process blood samples and analyse capillary glucose, serum insulin, and plasma GLP-1 are provided in Additional file 3: Method 3.

### Calculations

The incremental area under the curve (iAUC) over the period from 0 to 120 min was calculated for glucose, insulin, and GLP-1 according to the trapezoidal rule, ignoring the area below fasting values [32]. The maximal concentration (C_max_) and time to reach maximal concentration (T_max_) for glucose and insulin responses and peak antrum area were determined using a non-compartmental method.

### Statistical analyses and sample size calculations

The normality of distribution of all outcome variables was visually inspected by histograms. Highly skewed variables (glucose iAUC_2h_, GER, *T*_*1/2*_, GLP-1 iAUC_2h_) were log-transformed to normalise the distribution. Variables are presented as means ± standard deviations, and log-transformed variables as geometric means with 95% confidence intervals. Treatment effect was estimated by applying a linear mixed-effects model with treatment as fixed effects and subjects as random effects. Random intercepts and random slopes for each subject were included in the models to adjust for intra-subject variability. The linear mixed-effects modelling was performed in R 4.1.3 (R Core Team, 2022) using the packages lme4 [33] and nlme [34]. Descriptive statistics were performed in IBM® SPSS Statistics for Macintosh, version 26 (Armonk, NY: IBM Corp.). All tests were two-tailed with a significance level of 0.05.

A sample size of 22 was required to detect a 25 min difference in gastric emptying *T*_*1/2*_ with α set at 0.05 at a power of 0.80. The power calculation is based on standard deviations obtained from Thondre et al. [20], where *T*_*1/2*_ was estimated after consumption of a soup containing 12.9 g added high-MW β-glucan (SD = 43.9) compared with consumption of a control soup without β-glucan (SD = 11.2) in a crossover trial. To account for an estimated 10% dropout rate, 25 subjects were randomised.

## Results

### Subject characteristics

**Fig. 1:**
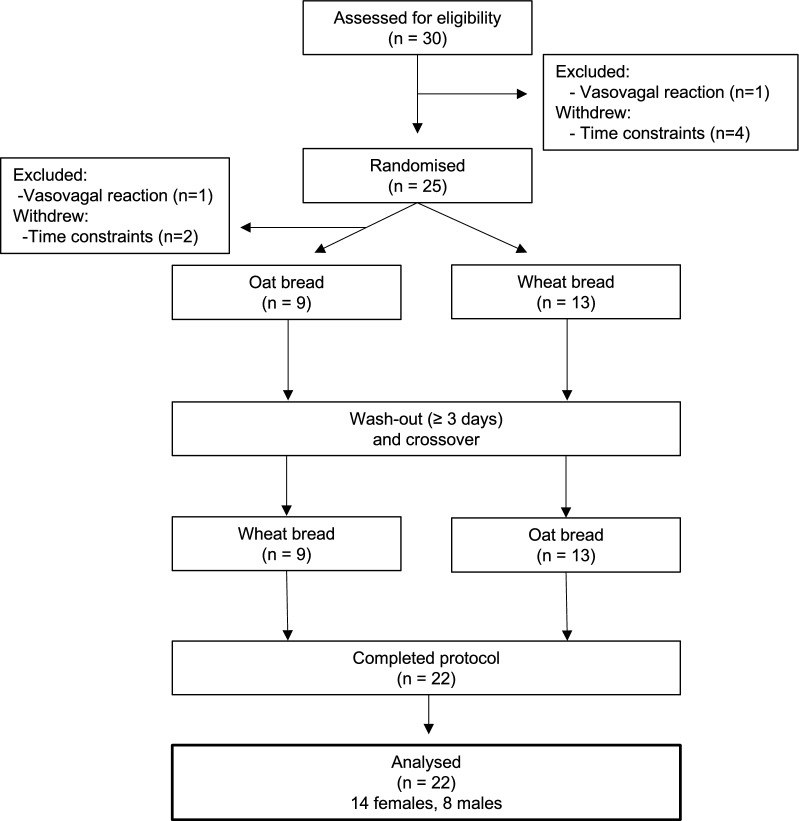
Study flowchart showing study design and number of recruited, excluded, included, and randomised participants

Thirty individuals were assessed for eligibility. Four individuals withdrew from the study before, and two after, the allocation process due to time constraints, whereas one individual was excluded before and one individual after the allocation process due to vasovagal reactions (Fig. 1). The anthropometric and biochemical characteristics of the 22 subjects that completed the trial according to protocol are presented in Table 2. Baseline characteristics of the subject allocated to the oat bread versus the wheat bread in the first period is shown in Additional file 5: Table S2.

**Table 2:** Anthropometric and biochemical baseline characteristics of the 22 healthy adults completing the study according to protocol

Variable	Total (n = 22)	Males (n = 8)	Females (n = 14)
Sex (males/females), n	8/14	8	14
Age (years)	24.6 ± 3.1	24.9 ± 2.3	24.5 ± 3.6
Weight (kg)	71.1 ± 13.8	82.9 ± 13.9	64.3 ± 8.3
BMI (kg/m^2^)	23.1 ± 2.7	24.2 ± 2.5	22.4 ± 2.7
WC (cm)	77.8 ± 9.5	85.6 ± 8.2	73.4 ± 7.2
Systolic BP (mmHg)	111.1 ± 65.1	119.0 ± 13.5	106.6 ± 5.5
Diastolic BP (mmHg)	65.1 ± 8.8	64.2 ± 10.7	65.6 ± 8.0
Fasting capillary blood glucose (mmol/L)	4.9 ± 0.7^1^	5.3 ± 0.9	4.8 ± 0.5^1^
Fasting insulin (mlU/L)	5.2 ± 2.6	6.4 ± 2.7	4.6 ± 2.5
HbA1c (mmol/mol)	30.2 ± 3.9	30.9 ± 3.3	29.8 ± 4.3

Values are expressed as mean ± standard deviation or n. ^1^Fasting capillary blood glucose was missing for one subject and thus replaced by fasting plasma glucose (converted according to recommendations by applying a factor of 1.11 ([whole blood glucose/capillary glucose] = [plasma glucose]/1.11) [35]. *BMI* body mass index; *BP* blood pressure; *HbA1c* glycated haemoglobin; *WC* waist circumference

### Gastric emptying

**Fig. 2:**
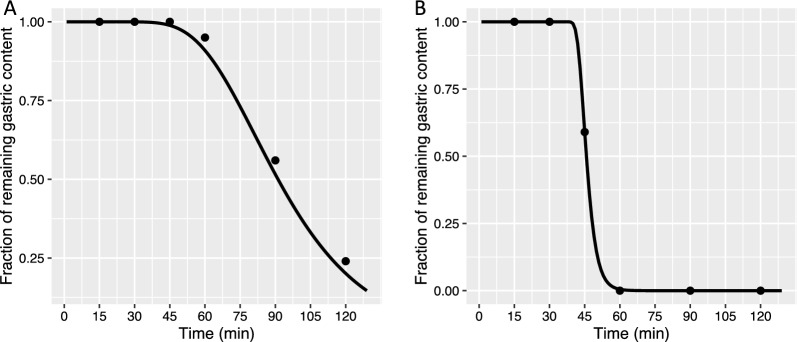
Individual gastric emptying curves after consumption of β-glucan-enriched oat bread (**A**) and whole-wheat bread (**B**). The figures show typical examples of individual gastric emptying curves fitted to a non-linear modified Elashoff power exponential equation y(t) = 1 − (1 − exp^−kt^)^β^ describing biphasic emptying. The emptying curves are from the same participant

The ingestion times of the oat bread and wheat bread were 7.1 ± 2.5 min and 6.6 ± 2.2 min, respectively, with no statistically significant difference between the treatments (*p*-value obtained from a paired sample t-test = 0.290). The gastric emptying profile for the majority of subjects for both breads displayed a biphasic pattern with a distinct *T*_*lag*_ before the commencement of a power exponential phase of emptying (Fig. 2). The mean R^2^ value of the individual gastric emptying profiles fitted to the modified power exponential equation was 0.93 with a standard deviation of 0.09.

The consumption of the oat bread was found to increase *T*_*1/2*_ and *T*_*lag*_ compared with the wheat bread (78.9 (67.2, 92.6) vs. 61.9 (53.5, 71.8) min, *p* = 0.005 and 63.3 ± 26.1 vs. 49.1 ± 19.6 min, *p* = 0.010, respectively), whereas no differences were observed for GER (rate constant *k* = 0.05 (0.03, 0.07) vs. 0.05 (0.04, 0.08) min^−1^ after consumption of the oat bread and wheat bread, respectively, *p* = 0.562). Peak antrum CSA was higher after consumption of the oat bread compared with the wheat bread (7.10 ± 1.32 vs. 6.40 ± 1.37 cm^2^, *p* = 0.039).

### GLP-1

**Fig. 3:**
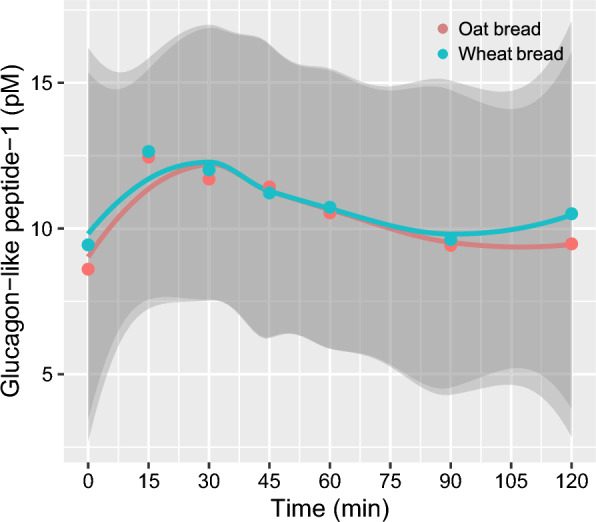
Mean postprandial glucagon-like peptide-1 response after consumption of β-glucan-enriched oat bread and whole-wheat control bread. Mean postprandial response of the 22 healthy adults completing the crossover study according to protocol. Shaded areas are 95% confidence bands. Points are mean values. There was no statistically significant difference in GLP-1 response between treatments

There were no differences in iAUC_2h_ for the postprandial GLP-1 response after consumption of oat bread compared with wheat bread (139 (72, 268) vs. 50 (17, 151) pM*min, respectively, *p* = 0.892, Fig. 3).

### Glycaemic and insulinemic responses

**Fig. 4:**
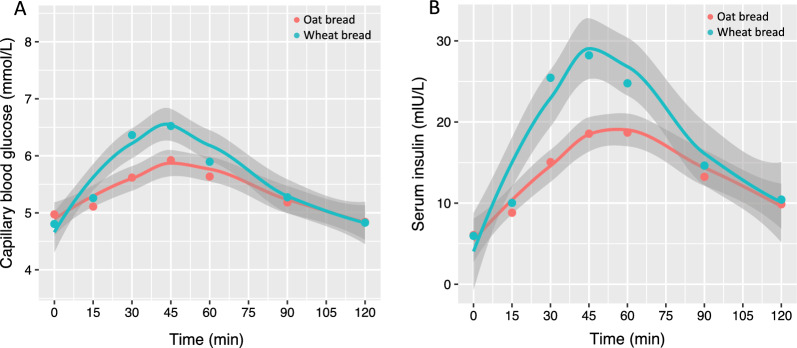
Mean postprandial capillary blood glucose (**A**) and serum insulin (**B**) response. Mean postprandial responses after consumption of a β-glucan-enriched oat bread compared with a whole-wheat control bread in the 22 healthy adults completing the crossover study according to protocol. Shaded areas are 95% confidence bands. Points are mean values. Postprandial glycaemic and insulinemic responses, assessed as 2-h incremental area under the curve, were statistically significant different between treatments (*p* < 0.001 for both)

The postprandial glycaemia, assessed as iAUC_2h_, and C_max_ for glucose response was significantly lower after consumption of the oat bread compared with the wheat bread (38.9 (22.9, 66.1) vs. 87.1 (65.4, 115.9) mmol/L*min, *p* = 0.001, and 6.2 ± 0.7 vs. 6.9 ± 0.9 mmol/L, *p* < 0.001, respectively, Fig. 4). There was no difference in T_max_ for glucose response after consumption the oat bread compared with the wheat bread (42.3 ± 18.7 vs. 42.3 ± 11.0 min, *p* > 0.99).

The postprandial insulinemic response, assessed as iAUC_2h_, was significantly lower after consumption of the oat bread compared with the wheat bread (938 ± 440 vs. 1440 ± 722 mlU/L*min, *p* < 0.001, Fig. 4). Consumption of the oat bread compared with the wheat bread led to significantly lower C_max_ for the insulin response (21.3 ± 9.4 vs. 32.0 ± 17.7 mLU/L, *p* = 0.001), but no difference was observed for T_max_ (49.8 ± 18.2 vs. 51.1 ± 21.0 min, *p* > 0.99).

## Discussion

Contrary to expectations, differences after consumption of the β-glucan-enriched oat bread compared with the whole-wheat bread were only seen for *T*_*1/2*_ and *T*_*lag*_, not for GER or GLP-1 response, even though the anticipated differences in postprandial glycaemia and insulinemia were evident.

The similar GER observed after consumption of the oat bread compared with the wheat bread supports the theory that once size of the solids becomes small enough, the stomach content will empty at the same rate [13]. β-glucan’s ability to increase viscosity in the stomach, making the time to break down the stomach content into smaller particles longer, might explain our observed differences in *T*_*1/2*_ and *T*_*lag*_. MW and solubility/hydration are fundamental for the viscous properties of β-glucans, and high-MW β-glucans, which was applied in the present study (MW of 1050kD, Table 1), have been shown to attenuate postprandial glycaemia along with increased *T*_*1/2*_ and *T*_*lag*_ compared with low-MW β-glucans [20, 21].

There was a crude difference of 3.5 g protein between the oat and wheat bread applied in the present study (Table 1). Studies show inconsistent results regarding the effects of protein on gastric emptying, potentially reflecting differences in study protocols and methods used to assess gastric emptying [36–39]. An effect of protein load on gastric emptying has been found [40, 41]. By applying whey protein loads of 70 g compared with 14 g, an increase in *T*_*1/2*_ of 35 min [40] and 52 min [41] has been observed. Thus, a crude difference of 56 g whey protein influenced gastric emptying [40, 41], which is a protein difference 16 times that in the present study. Interestingly, consumption of whey protein has been found to slow gastric emptying compared with consumption of casein, cod, and gluten proteins [38], which is of relevance for the present study as the study breads contain gluten proteins. Though increasing loads of protein has been found to slow gastric emptying, this is suggested to be related to difference in energy intake when consuming different protein loads [36, 37]. Thus, gastric emptying is suggested to be independent of protein load [36, 37]. Although the oat bread contained 3.5 g more protein than the wheat bread in the present study, it is unlikely that this marginal disparity could explain the observed differences in gastric emptying *T*_*1/2*_ and *T*_*lag*_. This might also be reflected by the insulinotropic effects of dietary proteins [42]. Despite the protein content being higher in the oat bread, the postprandial insulinemic response was significantly lower after consumption of the oat bread compared with the wheat bread.

Moreover, as higher energy consumption delays gastric emptying [12, 43], the increased *T*_*1/2*_ observed after consumption of the oat bread could reflect the serving of oat bread containing approx. 12% more energy than the wheat bread (corresponding to a 83 kJ [20 kcal] difference, Table 1). However, the difference in energy content in our study was much smaller than for the high-energy meal found to delay gastric emptying by Camps et al. [43] as this meal contained 400% more energy than the low-energy meal (corresponding to a 1674 kJ (400 kcal) difference). The high-energy meal increased the *T*_*1/2*_ by ~ 41 min compared with the low-energy meal [43], which extrapolated to our results would yield a ~ 1 min increased *T*_*1/2*_ based on the difference in energy load, which confers to be an unlikely explanation for the difference in *T*_*1/2*_. Of note, though Camps et al. [43] found energy load to confer a more pronounced effect, they also found meals with higher viscosity to increase *T*_*1/2*_.

The peak antrum area was measured to be slightly larger after consumption of the oat bread compared with the wheat bread, which might be due to a more rapid liquid emptying after consumption of the wheat bread whereas the β-glucans in the oat bread might have mixed with the water increasing the gastric volume. However, ultrasonography cannot readily differentiate between emptying of solids and liquids, and thus cannot provide information whether the water consumed with the bread mixed with the chyme in the stomach or funnelled from the fundus directly to the duodenum [44]. Further, secretion of gastric juices naturally increases after food consumption, and meal viscosity has been found to stimulate the secretion even more [12]. Together, the larger peak antrum area after consumption of the oat bread may reflect the serving of oat bread having a higher weight than the wheat bread (crude difference 34.1 g of which 25.1 g was moisture), and the viscous properties of β-glucans increasing gastric secretions, binding to the water consumed, and increasing the time needed to break down the stomach content before gastric emptying (e.g., *T*_*lag*_). Since no differences in time spent to consume the two bread meals were detected in the present study, variations in ingestion times are an unlikely explanation for the differences observed for *T*_*1/2*_ and *T*_*lag*_.

Due to the increased *T*_*1/2*_ and *T*_*lag*_ after consumption of the oat bread compared with the wheat bread, one could expect a later peak in GLP-1 secretion after consumption of the oat bread, but no differences in the postprandial period were detected. An increase in GLP-1 levels at 120 and 150 min postprandially after consumption of a rye bread containing 5.4 g β-glucans has been demonstrated [45]. Thus, effects on later phase GLP-1 secretion cannot be excluded, but this was not measured in the present study.

The present study supports the ability of a β-glucan-enriched bread to lower glycaemic and insulinemic responses independently of the effect on GER. Further, it shows that the biphasic nature of stomach emptying, e.g., *T*_*lag*_, is important to consider. The food matrix is also an aspect to contemplate as this might influence bolus formation, susceptibility to disintegration in the stomach and the subsequent rate of β-glucan hydration, and thus viscosity development in the stomach. The high moisture content and the continuous distribution of β-glucans throughout the oat bread applied in the present study are likely to give a fast increase in viscosity of the whole stomach content. This might not be the case when β-glucans only are present as a dry constituent, e.g., granola, of a meal, though the amount and MW of β-glucans are similar. Thus, the food matrix may influence the physicochemical properties of β-glucans, which in turn may affect the physiological responses, such as postprandial glycaemia.

Strengths of the present study include the use of an oat bread developed to contain high amounts of high-MW β-glucan and the inclusion of a method considering the biphasic nature of stomach emptying by fitting individual emptying profiles to a modified power exponential curve [30, 31].

Limitations which may be relevant include the mismatch in weight, energy, and protein content of the two study breads, the lack of blinding of study personnel due to practical reasons, and the lack of a standardised evening meal to avoid the second meal effect which might affect postprandial metabolic responses [46], though the latter can be debated [47]. Though participants were blinded, it cannot be excluded that they were able to distinguish the two bread meals based on appearance, smell, and taste, which is a general limitation in nutritional intervention studies. Moreover, measuring the gastric antrum area immediately following the bread intake may have yielded additional information on the gastric emptying process. The method applied to estimate gastric emptying could not distinguish between emptying of liquids versus solids, nor was it possible to estimate the total gastric volume, which could have provided additional information on the gastric emptying process. Lastly, as gastric emptying has been found to differ between menstrual phases, being slower during the luteal phase compared with the follicular phase [48], another limitation of the study is not including measures of female sex hormones to be able to distinguish the menstrual phases. However, others have not found any influence of the menstrual phases on gastric emptying [49].

In conclusion, consumption of a β-glucan-enriched oat bread increased gastric emptying half-time and lag phase compared with a whole-wheat bread, whereas no differences were found for gastric emptying rate or GLP-1 response. Thus, a plausible mechanism for the observed attenuation of the postprandial glycaemic and insulinemic responses after oat bread consumption might be attributed to β-glucans’ ability to rapidly increase viscosity of the stomach content. Based on the effect of the β-glucan-enriched oat bread on the glycaemic response in healthy adults, future studies should investigate whether people with impaired glucose metabolism share the same beneficial response.

## Supplementary Information

**Additional file 1. Method 1.** Nutrient composition analyses.

**Additional file 2. Method 2.** Procedures for measuring blood pressure and anthropometrics.

**Additional file 3. Method 3.** Biochemical analyses.

**Additional file 4. Table S1. **Ingredients for production of the β-glucan-enriched oat bread and the whole-wheat bread applied in a crossover trial assessing potential mechanisms for the glucose-lowering abilities of β-glucans.

**Additional file 5, Table S2.** Anthropometric and biochemical baseline characteristics of 22 healthy, young adults receiving a β-glucan enriched oat bread and a whole wheat bread at two separate occasions with at least a three-day washout in-between.

## Data Availability

Data described in the manuscript and analytic code will be made available upon request pending on individual consideration. Requests require approval of the application and use of the data by the project group and by the Regional Committee for Medical and Health Research Ethics West, Norway, who will issue ethical approval for the request. A signed data-sharing agreement is required. The data will not be freely available due to participant confidentiality. Informed consent was not obtained for publication of participant data.

## Abbreviations

C_max_: Maximum concentration
GER: Gastric emptying rate
GLP-1: Glucagon-like peptide 1
iAUC: Incremental area under the curve
MW: Molecular weight
*T*_*lag*_: Duration of gastric emptying lag phase
T_max_: Time to reach maximum concentration
*T*_*1/2*_: Gastric emptying half time
