# Supplementary material for: The acute effect of a β-glucan-enriched oat bread on gastric emptying, GLP-1 response, and postprandial glycaemia and insulinemia: a randomised crossover trial in healthy adults
Source: Nutr Metab (Lond). 2024 Mar 18;21:13. doi: 10.1186/s12986-024-00789-w (PMC10949669; doi:10.1186/s12986-024-00789-w)
Supplement: Supplementary file 1 — Additional file 1. Method 1. Nutrient composition analyses. [file 12986_2024_789_MOESM1_ESM.docx]

# **Supplementary Method 1.** Nutrient composition analyses

Protein was estimated from the analysis of total N by combustion (1) using a Vario EL elemental analyser (Elementar, Langenselbold, Germany) with a nitrogen to protein conversion factor of 5.7 (2). Fat was determined gravimetrically following acid hydrolysis, extraction into diethyl ether and petroleum ether followed by evaporation. Total dietary fibre was determined according to the gravimetric method of AOAC 985.28. Total cereal β-glucan was determined by enzymatic assay using a Megazyme (Brey, Ireland) mixed-linkage β-glucan kit according to AOAC 995.16. Sugars were determined as the sum of sucrose, glucose and fructose by analysis with anion-exchange chromatography with pulsed amperometric detection following their extraction from bread in 50% water:methanol. Total and resistant starch was determined by AOAC 2002.02. Available CHO was subsequently calculated as described by Brouns et al. (3). Bulk water content was determined gravimetrically by freeze-drying followed by subsequent final drying at 103 °C to constant weight. Ash was determined gravimetrically as the inorganic residue (mineral content) remaining after removal of all water and organics by combustion in a muffle furnace at 550 °C overnight. Total energy content was calculated according to EU Council Directive 1169/2011. Weight-average molar mass of oat β-glucan in bread was determined by size-exclusion chromatography with multi-angle light scattering as described for galactomannan by Mæhre et al. (4). All measurements were made at least in duplicate.

References:

1. Kirsten WJ. Automatic Methods for the Simultaneous Determination of Carbon, Hydrogen, Nitrogen, and Sulfur, and for Sulfur Alone in Organic and Inorganic Materials. Anal Chem. 1979;51(8):1173–9.
2. Mariotti F, Tomé D, Mirand PP. Converting nitrogen into protein - Beyond 6.25 and Jones’ factors. Crit Rev Food Sci Nutr. 2008 Feb;48(2):177–84.
3. Brouns F, Bjorck I, Frayn KN, Gibbs AL, Lang V, Slama G, et al. Glycaemic index methodology. Nutr Res Rev. 2005 Jun;18(1):145–71.
4. Mæhre, H. K., Weisensee, S., Ballance, S., Rieder, A. (2021). Guar gum fortified breads for prospective postprandial glycaemic control – Effects on bready quality and galactomannan molecular weight. LwT 152, 112354.
