# Supplementary material for: The acute effect of a β-glucan-enriched oat bread on gastric emptying, GLP-1 response, and postprandial glycaemia and insulinemia: a randomised crossover trial in healthy adults
Source: Nutr Metab (Lond). 2024 Mar 18;21:13. doi: 10.1186/s12986-024-00789-w (PMC10949669; doi:10.1186/s12986-024-00789-w)
Supplement: Supplementary file 2 — Additional file 2. Method 2. Procedures for measuring blood pressure and anthropometrics. [file 12986_2024_789_MOESM2_ESM.docx]

# **Supplementary Method 2.** Procedures for measuring blood pressure and anthropometrics.

Blood pressure was determined by using a digital blood pressure monitor (Omron model HEM-907, Omron Healthcare Asia). Participants were instructed to rest for 5 minutes in an upright, seated position before the first of three measurements with one-minute intervals was conducted. The mean of the two last measurements was used as an estimate of the blood pressure.

Body weight was measured to the nearest 0.1 kg without shoes in light clothing using a digital scale (Seca Mode 877, Hamburg, Germany). Height was measured only at the first attendance to the nearest 0.1 cm by using a portable stadiometer (Seca Mode 217, Hamburg, Germany). The participants were instructed to have their feet gathered, arms along the side, relaxed shoulders, and their head in the Frankfurt horizontal plane.

Waist circumference was determined in a standing position at the midpoint between the iliac crest and the lowest palpable rib margin on the bare skin to the nearest 0.1 cm using a non-stretch tape measurer (Seca Mode 201, Hamburg, Germany).
