# Supplementary material for: The acute effect of a β-glucan-enriched oat bread on gastric emptying, GLP-1 response, and postprandial glycaemia and insulinemia: a randomised crossover trial in healthy adults
Source: Nutr Metab (Lond). 2024 Mar 18;21:13. doi: 10.1186/s12986-024-00789-w (PMC10949669; doi:10.1186/s12986-024-00789-w)
Supplement: Supplementary file 3 — Additional file 3. Method 3. Biochemical analyses. [file 12986_2024_789_MOESM3_ESM.docx]

# **Supplementary Method 3.** Biochemical analyses.

Capillary blood was collected by finger prick samples and glucose concentrations were determined by a modified glucose dehydrogenase method using a photometer (HemoCue Gucose 201 RT Analyzer, HemoCue, Sweden).

For determination of serum insulin, venous blood samples were collected in vacutainers with serum separator gel and clot activators (BD Vacutainer® SST^TM^ Advanced, ref. 367955). The samples were allowed to clot for 30 min at room temperature before centrifuged at 2200 G for 10 min at 20 °C. The serum samples were stored at -80 °C until analysed at the Department of Medical Biochemistry and Pharmacology, Helse Bergen (accreditation ref. NS-EN ISO 15189:2012) using chemiluminescent immunoassay (Siemens Healthineers Immunolite 1000 XPi, Siemens Healthcare GmbH, Germany).

For determination of GLP-1, blood samples were collected in vacutainers with K_2_EDTA (BD Vacutainer® Plus Plastic K_2_EDTA Tubes, ref 367842). To avoid inactivation of GLP-1, Dipeptidyl peptidase-IV inhibitors (10 uL/mL) were added to the vacutainers before sampling. The vacutainers were placed on ice before and after the blood collection, and centrifuged within 20 min after sampling at 2000 G for 10 min at 4°C. The samples were stored at -80 °C until analysed with the commercial kit Glucagon-Like Peptide 1 (Active) ELISA kit (EGLP-35K, Merck KGaA, Darmstadt, Germany) according to the manufacturer’s protocol. The plates were read in room temperature using SPECTRA max GeminiEM Microplate Fluorescence reader (Molecular Devices, Sunnyvale, CA, USA) with an excitation/emission wavelength of 355 nm/460 nm.
