# Supplementary material for: The acute effect of a β-glucan-enriched oat bread on gastric emptying, GLP-1 response, and postprandial glycaemia and insulinemia: a randomised crossover trial in healthy adults
Source: Nutr Metab (Lond). 2024 Mar 18;21:13. doi: 10.1186/s12986-024-00789-w (PMC10949669; doi:10.1186/s12986-024-00789-w)
Supplement: Supplementary file 4 — Additional file 4. Table S1. Ingredients for production of the β-glucan-enriched oat bread and the whole-wheat bread applied in a crossover trial assessing potential mechanisms for the glucose-lowering abilities of β-glucans. [file 12986_2024_789_MOESM4_ESM.docx]

**Supplementary Table 1.** Ingredients for production of the β-glucan-enriched oat bread and the whole-wheat bread applied in a crossover trial assessing potential mechanisms for the glucose-lowering abilities of β-glucans.

| **Ingredients** | **Supplier** | **Oat bread (%)** | **Wheat bread (%)** |
| --- | --- | --- | --- |
| Rapeseed oil | Idun Industri AS, Norway | 0.7 | 4.7 |
| Dry yeast | Idun Industri AS, Norway | 0.7 | 0.6 |
| Salt | GC Rieber AS, Norway | 1.0 | 1.0 |
| Sieved white wheat flour | Lantmännen Cerealia, Norway | 21.9 | 18.7 |
| Wholegrain wheat flour | Lantmännen Cerealia, Norway | 0 | 37.5 |
| Water | Oslo kommune, Norway | 53.8 | 37.5 |
| SWEOAT® Bran BG14 Bakery | Swedish Oat fiber, Sweden | 21.9 | 0 |
