# Supplementary material for: The acute effect of a β-glucan-enriched oat bread on gastric emptying, GLP-1 response, and postprandial glycaemia and insulinemia: a randomised crossover trial in healthy adults
Source: Nutr Metab (Lond). 2024 Mar 18;21:13. doi: 10.1186/s12986-024-00789-w (PMC10949669; doi:10.1186/s12986-024-00789-w)
Supplement: Supplementary file 5 — Additional file 5, Table S2. Anthropometric and biochemical baseline characteristics of 22 healthy, young adults receiving a β-glucan enriched oat bread and a whole wheat bread at two separate occasions with at least a three-day washout in-between. [file 12986_2024_789_MOESM5_ESM.docx]

**Supplementary Table 2.** Anthropometric and biochemical baseline characteristics of 22 healthy, young adults receiving a β-glucan enriched oat bread and a wholewheat bread at two separate occasions with at least a three-day washout in-between.

| **Variable** | **Total (n=22)** | **Oat bread first (n=9)** | **Wheat bread first (n=13)** |
| --- | --- | --- | --- |
| **Sex (males/females), n** | 8/14 | 4/5 | 4/9 |
| **Age (years)** | 24.6 ± 3.1 | 24.3 ± 3.5 | 24.9 ± 3.0 |
| **Weight (kg)** | 71.1 ± 13.8 | 70.3 ± 11.4 | 71.6 ± 15.6 |
| **BMI (kg/m^2^)** | 23.1 ± 2.7 | 22.5 ± 2.4 | 23.5 ± 2.9 |
| **Waist circumference (cm)** | 77.8 ± 9.5 | 77.0 ± 8.5 | 78.3 ± 10.5 |
| **Systolic BP (mmHg)** | 111.1 ± 65.1 | 112.9 ± 11.8 | 109.9 ± 10.4 |
| **Diastolic BP (mmHg)** | 65.1 ± 8.8 | 68.2 ± 10.0 | 63.0 ± 7.6 |
| **Fasting capillary blood glucose (mmol/L)** | 4.9 ± 0.7^1^ | 5.2 ± 0.9 | 4.7 ± 0.5^1^ |
| **Fasting insulin (mlU/L)** | 5.2 ± 2.6 | 5.7 ± 2.5 | 4.9 ± 2.8 |
| **HbA1c (mmol/mol)** | 30.2 ± 3.9 | 30.1 ± 3.9 | 30.2 ± 4.1 |
|  |  |  |  |

Values are expressed as mean ± SD or *n*.
^1^Fasting capillary blood glucose was missing for one subject and thus replaced by fasting plasma glucose (converted according to recommendations by applying a factor of 1.11 ([whole blood glucose/capillary glucose] = [plasma glucose]/1.11) (ref. 1).
BMI, body mass index; BP, blood pressure; HbA1c, glycated haemoglobin; SD, standard deviation.

**Reference:**

1. D’Orazio P, Burnett RW, Fogh-Andersen N, Jacobs E, Kuwa K, Külpmann WR, et al. Approved IFCC recommendation on reporting results for blood glucose (abbreviated). Clin Chem. 2005 Sep;51(9):1573–6.
